# Three-Dimensional Columnar Microstructure Representation Using 2D Electron Backscatter Diffraction Data for Additive-Manufactured Haynes^®^282^®^

**DOI:** 10.3390/ma17071659

**Published:** 2024-04-04

**Authors:** Liene Zaikovska, Magnus Ekh, Johan Moverare

**Affiliations:** 1 Department of Engineering Science, University West, SE-461 86 Trollhättan, Sweden; johan.moverare@liu.se; 2 Department of Material and Computational Mechanics, Chalmers University of Technology, SE-412 06 Gothenburg, Sweden; magnus.ekh@chalmers.se; 3 Department of Management and Engineering, Linköping University, SE-581 83 Linköping, Sweden

**Keywords:** PBF-EB, EBSD, polycrystal, anisotropy, RVE, computational homogenization

## Abstract

This study provides a methodology for exploring the microstructural and mechanical properties of the Haynes^®^282^®^ alloy produced via the Powder Bed Fusion-Electron Beam (PBF-EB) process. Employing 2D Electron Backscatter Diffraction (EBSD) data, we have successfully generated 3D representations of columnar microstructures using the Representative Volume Element (RVE) method. This methodology allowed for the validation of elastic properties through Crystal Elasticity Finite Element (CEFE) computational homogenization, revealing critical insights into the material behavior. This study highlights the importance of accurately representing the grain morphology and crystallographic texture of the material. Our findings demonstrate that created virtual models can predict directional elastic properties with a high level of accuracy, showing a maximum error of only ~5% compared to the experimental results. This precision underscores the potential of our approach for predictive modeling in Additive Manufacturing (AM), specifically for materials with complex, non-homogeneous microstructures. It can be concluded that the results uncover the intricate link between microstructural features and mechanical properties, underscoring both the challenges encountered and the critical need for the accurate representation of grain data, as well as the significance of achieving a balance in EBSD area selection, including the presence of anomalies in strongly textured microstructures.

## 1. Introduction

Additive Manufacturing (AM) technologies have been attractive and of high interest in recent years. The flexibility of producing complex metal components allows increased design performance and structural optimization. Powder Bed Fusion-Electron Beam (PBF-EB) and laser beam melting, referred to as Powder Bed Fusion-Laser Beam (PBF-LB), are two of the AM techniques classified as powder bed processes. This study focuses on the PBF-EB process, which utilizes a high-energy electron beam to selectively melt and fuse metal powder particles, layer by layer, to create complex three-dimensional metal parts. The PBF-EB process offers several advantages, including the ability to produce fully dense metal parts with complex geometries, excellent mechanical properties, and a high degree of design flexibility. It is particularly suitable for manufacturing parts with high melting points, such as superalloys [1] and titanium alloys [2], used in aerospace [3], medical [4], and automotive industries [5]. The formation of a columnar microstructure, parallel with the build direction (BD), is characteristic of the grain structure produced through PBF-EB. The PBF-EB technology is applicable to several different materials; nevertheless, each material requires tweaking and identifying optimal processing parameters, which might not always be straight forward. However, with optimized parameter control established, it produces a promising tailored microstructure with 99.5% dense material and no observable cracking [6].

This study involves the use of the Haynes^®^282^®^ alloy, a recently developed γ′-strengthened Ni-based superalloy commonly used in high-temperature applications, including gas turbine components, such as combustion chambers and exhaust systems. It finds application in aerospace, power generation, and other industries that require materials capable of withstanding extreme temperatures and demanding operating conditions [6,7]. The exceptional properties of Haynes^®^282^®^ make it a preferred choice for critical applications where reliability, durability, and high-temperature performance are essential. Haynes^®^282^®^ demonstrates excellent thermal stability, meaning it retains its mechanical properties and dimensional stability even when exposed to thermal cycling or fluctuations in temperature. This work introduces a representative method to mimic and virtually test the microstructure of Haynes^®^282^®^, aiming to establish comprehensive connections between the material’s structure and its properties. The most difficult challenge in material characterization lies in determining a suitable representation for the heterogeneous and anisotropic material, which is essential for conducting virtual testing to predict the mechanical properties at the macroscale level. This is this paper’s contribution to the research field.

Electron Backscatter Diffraction (EBSD) is a Scanning Electron Microscopy (SEM) technique [8] that can be used to identify the crystallographic texture, which, in this study, was used as an input to the 3D RVE model generation. EBSD is a powerful microscopy technique used to study the materials at the micro- and nanoscales. It utilizes an electron beam to interact with a sample surface, and by analyzing the patterns formed by the backscattered electrons, it provides information about the crystal structure, grain orientation, grain boundaries, and other microstructural features of the material. An electron beam is focused onto the sample surface and interacts with the atoms in the sample, generating backscattered electrons. The backscattered electrons diffract as they interact with the crystal lattice of the material and can be used to determine the orientation and crystallographic information for each point on the sample. By scanning the electron beam across the sample, a series of diffraction patterns is collected, enabling the generation of orientation maps that reveal the distribution and boundaries of the individual grains within the material. The acquired EBSD data are further analyzed to extract information about grain boundaries, grain size, crystal defects, texture, and other microstructural features. It provides valuable insights into material properties, deformation mechanisms, phase transformations, and other aspects of material behavior.

Two critical microstructural parameters that strongly influence the mechanical properties of polycrystalline materials are the grain morphology and crystallographic texture. These parameters have undergone extensive investigation using a wide range of analytical tools [9]. In the process of generating grains, each grain is individually characterized by specific properties, with its mechanical and physical attributes being closely linked to their microstructure. It should also be noted that the distribution of grain sizes is of critical importance, particularly impacting what is referred to as “Hall–Petch strengthening”, which is closely associated with variations in the stress and strain within the material [10].

In recent years, the topic of grain formation and microstructure representation has gained prominence, underscored by the research presented in the study by Pauza et al. [11]. This research highlights the evolving understanding of how grains are formed and organized within materials, especially in the context of AM processes. By leveraging computer simulations, this study delves into the intricacies of microstructure development, focusing on the impact of the crystallographic texture. Such investigations are pivotal for advancing manufacturing techniques, enabling the production of materials with optimized properties tailored to specific applications. Several methods have been applied to mimic the actual microstructures. For instance, Vuppala et al. [12] discuss the methodology and considerations involved in extracting discrete orientation data from X-ray Diffraction (XRD) analysis for the purpose of accurately representing the texture of polycrystalline aggregates. Meanwhile, in a separate study [10], the researcher outlines the use of both statistical and XRD data, aiming to achieve an optimal polyhedral description of 3D polycrystals. In the study by Lizarazu et al. [13], the machine learning algorithms are employed to establish a relationship between the microstructural characteristics of AM mild steel and its mechanical properties, specifically the stress–strain curves. In another study by Calcagnotto et al. [14], both 2D and 3D EBSD techniques are utilized to explore the microstructural characteristics, especially orientation gradients and Geometrically Necessary Dislocations (GNDs), investigating the impact on the mechanical properties of ultrafine-grained dual-phase steels.

To enable the virtual testing of the AM material, computational homogenization is applied. It is a technique used to derive the effective macroscopic properties of a polycrystalline material from its microscopic structure. This method involves modeling the material subjected to external loads on a mesoscale and subsequently averaging the effects to predict the behavior of the material at the macroscale. Numerical methods [11,15], including the Crystal Elasticity Finite Element (CEFE) method [16,17], and the Crystal Plasticity Finite Element (CPFE) method [18] offer a sophisticated approach for the calculation of the homogenized mechanical properties. These simulations enable direct comparisons with experimental outcomes or can be utilized autonomously to methodically investigate the influence of microstructural features on material behavior. This depends upon the availability of the appropriate polycrystal models to enhance the ability to predict and optimize the properties of materials in AM processes.

One way to simulate the properties of polycrystalline microstructures is by using geometric models that offer conceptual simplicity and computational efficiency. Microstructures are often complex, featuring non-convex grain shapes that can only be described using voxel grids, which are not suitable for standard, conformal meshing. This complexity is also observed in the studies [19,20], where polycrystal images are obtained from EBSD data. In contrast, geometric models, like Voronoi or Laguerre tessellations, can be described in a concise, scalar manner using sets of points, lines, surfaces, and volumes [21,22]. A significant advantage is that they can be meshed using standard approaches, and the requirement of convex cells is often a minor limitation when representing polycrystals.

Biswas et al. [18] demonstrate considerable achievement in reflecting the actual microstructural characteristics, including texture and grain size. By employing large EBSD datasets for RVE generation, the study ensures the inclusion of a comprehensive range of microstructural information, thereby enhancing the representativeness and reliability of the simulations. Furthermore, the study suggests averaging results from multiple cropped EBSD datasets approximating the findings from larger datasets effectively, affirming the sufficiency of this combined approach for micromechanical modeling. Additionally, the studies conducted by Balanovskiy et al. [23,24] and Karlina et al. [25] show the important findings related to microstructure homogeneity and mechanical properties of AM materials, highlighting the potential for optimizing AM processes to achieve the desired material characteristics.

Our contribution to this field consists in a streamlined approach to accurately represent complex microstructures with minimal experimental input, leveraging 2D EBSD data to effectively simulate the properties of polycrystalline microstructures. By incorporating findings from the mentioned studies, we underline the importance of understanding and controlling microstructural anomalies and the influence of real-time process parameters on achieving homogeneity and optimal mechanical properties in AM-produced materials. This approach not only facilitates the broader adoption of these advanced manufacturing techniques in industrial applications but also contributes to sustainability by minimizing resource usage.

## 2. Materials and Methods

### 2.1. Experimental Procedure

In this study, the powder feedstock was Haynes^®^282^®^ powder produced via Sandvik Additive Manufacturing (Sandvik, Sweden) (https://www.home.sandvik/, accessed on 1 March 2024), with a special license from Haynes International (Kokomo, IN, USA). The metal powder is manufactured via vacuum induction melting with subsequent gas atomization using argon gas, resulting in a nominal powder particle size distribution of 45–106 µm. The chemical composition of the powder can be found in Table 1.

Furthermore, the PBF-EB samples were built using an Arcam A2X EBM machine (Mölndal, Sweden (https://www.ge.com/additive/, accessed on 1 March 2024). The manufacturing process commenced after pre-heating the powder bed to 1025 °C. The customized Arcam settings for Haynes^®^282^®^ were employed in this batch. Each deposition cycle included the following steps: preheating the current powder layer, contour melting of the build geometry, hatch melting of the interior of the build geometry, post-heating the current layer, and lowering the powder bed while raking the powders to create a uniform layer of approximately 70 µm for the next cycle. Table 2 presents the key process parameters implemented to minimize porosity and potential cracking.

**Figure 1:**
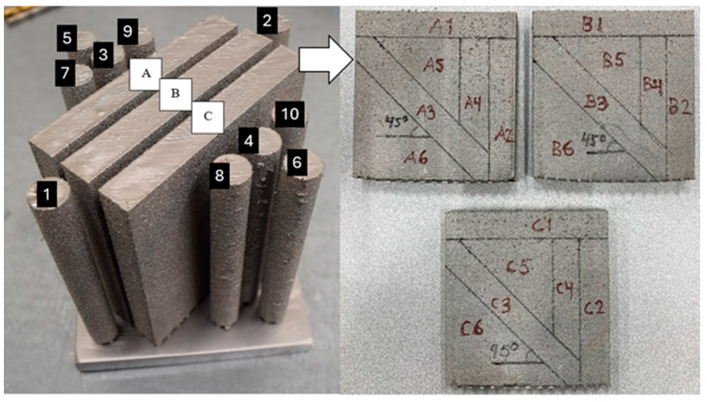
Sample geometries used for experimental tests. Cylinder 3 along with walls A, B, and C (100 × 100 × 10 mm) have been selected for the tensile specimen machining. The detailed notation for each wall is indicated by the white arrow. Specimens A1-4, B1-4 and C1-4 are selected for further investigation.

**Figure 2:**
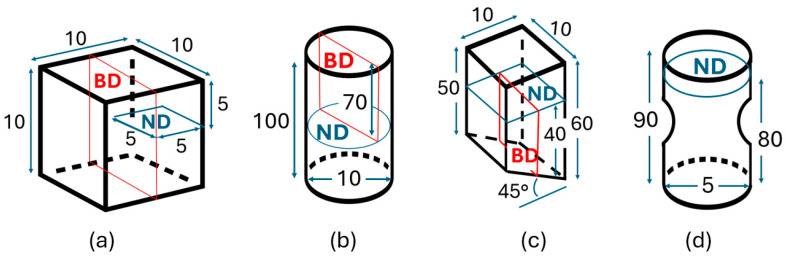
Schematic representation of EBSD segmentation in the build direction (BD) and normal direction (ND) planes for the (**a**) cube, (**b**) cylinder, (**c**) wall, and (**d**) test samples (unit: mm).

Three sample geometries, including a cube, a cylinder, and a wall, were produced using identical process parameters, as outlined in the preceding section. The dimensions of these geometries are depicted in Figure 1 and Figure 2. The cube represents the DoE sample demonstrating the most textured grain structure, having been chosen from a group of 16 varied process setting builds. This sample was not utilized for experimental tests nor for simulation validation. The cylinder labeled ‘3’, depicted in Figure 1, was chosen from a group of 10 cylinders for machining and subsequent testing. This specific cylinder was chosen for further study because of its placement and proximity to the walls. Additionally, for this investigation, three different walls labelled as A, B, and C were manufactured. From these wall structures, nine samples designated as 1 (H), 2 (V), and 3 (45°) were machined intended for the testing purpose. Furthermore, an extra wall sample, labelled as ‘A4’, ‘B4’, and ‘C4’, was produced from each wall to assess a larger area of the wall material for microstructure characterization. The sample cases below were employed for generating EBSD data and for case comparison, as illustrated in Figure 1:

- DoE sample, with case notation as ‘Cube’;
- Cylinder sample, with case notation as ‘Cylinder’;
- Wall samples 2 and 4 from each wall, with the case notations of ‘A2’, ‘B2’, and ‘C2’, and ‘A4’, ‘B4’, and ‘C4’, respectively;
- The notations ‘N1’, ‘N2’, and ‘N1R’ denote different EBSD maps from the same specimen.

In a prior investigation [27], it was demonstrated that the hatch region of a PBF-EB sample on Inconel 718 (Sandvik Additive Manufacturing, Sandvik, Sweden) exhibited a pronounced <001> crystallographic texture along the building direction. Conversely, the contour region of the sample did not display any obvious preferential crystallographic texture. Additionally, the study revealed that the volume fraction of the contour region was considerably smaller compared to the hatch region. For this study, test samples were initially utilized in their as-built condition. Nevertheless, these samples were subsequently machined from the cylinder and wall geometries to match the test sample shape, which was employed in the proceeding tests with samples machined from the hatch region.

Two types of tests, cyclic elasticity test and tensile test, were performed to determine the elastic modulus and generate the tensile stress–strain curve of the materials, respectively. To ensure the best possible alignment of the specimen and the accurate determination of the elastic modulus, the cyclic elasticity tests were performed using a servo hydraulic fatigue test rig with an Instron ±50 kN load cell, an Instron extensometer with a gauge length of 12.5 mm, and an Instron 8800 control system. The samples were exposed to 10 load cycles in the range of ±100 MPa, and the elastic modulus was calculated as the average slope of the stress vs. strain in the same range. A conventional tensile test was performed using an Instron 5582 universal test machine. All tests were performed in the open air and at room temperature. Given that cyclic elasticity tests yielded more accurate Young’s modulus results, they were selected for comparison and simulation validation.

### 2.2. RVE Generation

It is vital to ensure all samples are accurately polished and prepared, given their sensitivity; any surface scratches could present challenges during EBSD analysis as well as during the grain data tessellation. The samples designated for EBSD analysis were prepared using Bakelite and underwent mechanical grinding from 500 Grit to 4000 Grit. The polishing process involved diamond suspensions ranging from 3 to ¼ µm and concluded with OP-U colloidal silica suspension. The EBSD mappings were conducted using a Zeiss Gemini 450 SEM (Zeiss, Oberkochen, Germany), equipped with an EBSD system from Oxford Instruments (Abingdon, UK), operating at 15 kV.

The EBSD experimental technique delineates the raster polycrystal image at the voxel scale, specifying grain ID and lattice orientation. Grain IDs, coupled with the obtained lattice orientations, were employed to characterize grain morphology, encompassing details such as grain sizes, shapes, sample orientation, and crystallographic orientation.

Let M denote the set of all voxels, represented by $v_{k}$ for k=1,...,M, which describe the polycrystal structure. The identification of individual grains within this structure is achieved through the grain ID function, denoted as I. This function uniquely assigns each grain $G_{i}$ for i=1,...,N, whose specific identifier is as follows:

(1) $$G_{i}=\{v_{k}\in M|I(v_{k})=i\},$$

where Gi provides a comprehensive definition of the morphology and location of the grain within the domain [10]. Each voxel incorporates textured information as well, which is subsequently utilized to compute and gather the mean grain orientation for each grain derived from the raw EBSD data.

**Figure 3:**
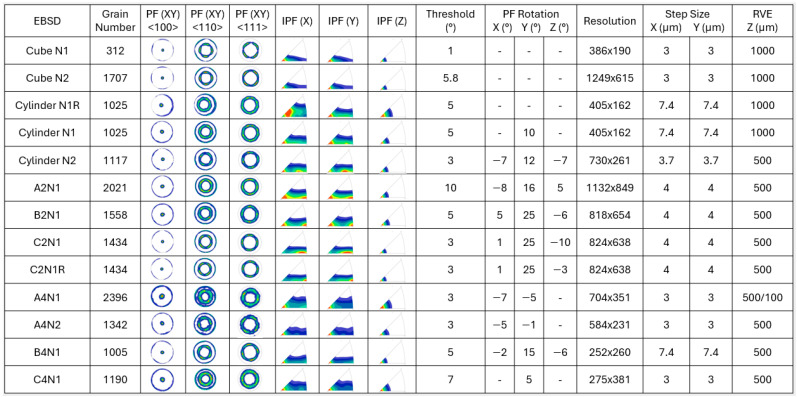
EBSD (Electron Backscattered diffraction) data specifications of the studied cases including grain number, pole figures (PFs), inverse pole figures (IPFs), threshold angle, PF rotational angle, resolution, step size and representative element volume (RVE) thickness.

The pixel data representing the microstructure image was read into the MATLAB 24.1.0 (R2024a) MTEX toolbox [28], where denoising and thresholding of grains were carried out, including the rotation of Pole Figure (PF) and Inverse Pole Figure (IPF) maps to align with the symmetrical results. This action was taken to improve image quality and facilitate the separation of individual grains, as well as the determination of mean grain orientations, with specific details provided in Figure 3. The grain segmentation was conducted using image processing algorithms to identify the individual grains. This step involves separating the connected regions in the image corresponding to different grains. The grain boundary extraction of each segmented grain was achieved using techniques like edge detection or region growing algorithms. The grain reconstruction was conducted by converting the segmented grain boundaries into a representative grain mesh and unique grain identification number.

**Figure 4:**
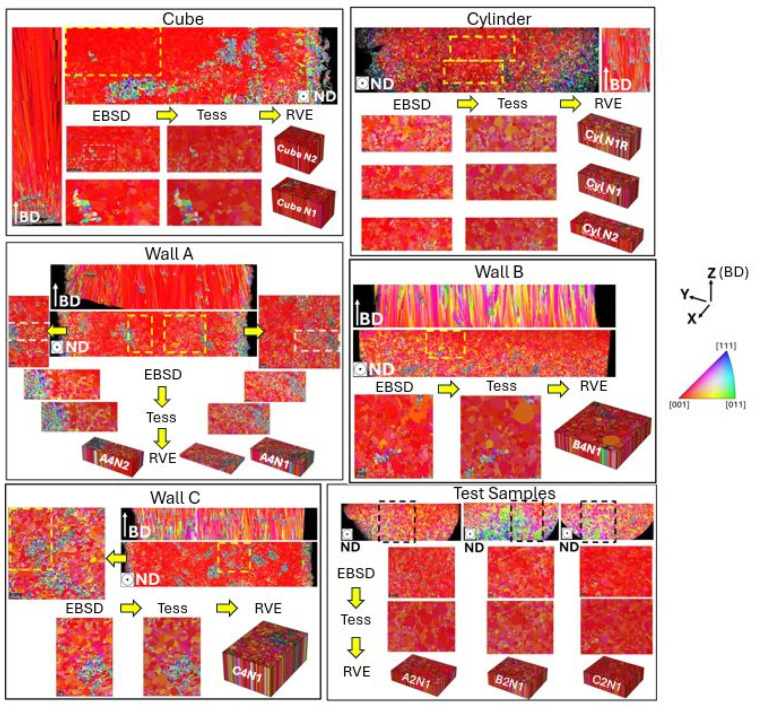
RVE representation of the cube, cylinder, wall, and test sample microstructures. The selected areas are marked with the yellow and black dotted lines.

The key input parameters are provided in Figure 3 to provide an overview of the setting for the studied cases. The number of grains for the EBSD sections was created within the range of 1000–2000 grains, with the exception of the case Cube N1, which included 312 grains. PF maps in <100>, <110>, <111> crystallographic directions, as well as IPF maps in plane normal to the x, y, z directions, are presented to illustrate the symmetry and comparison of the texture development within the grains. The threshold angle is crucial for defining grain boundaries, which subsequently determines the number of grains within a given EBSD area. With more non-convex shapes present, a higher threshold angle is required. However, it is important to note that an increased threshold angle leads to a more approximate representation of grain shapes and mean grain orientation. This factor significantly impacts the equilibrium between defining the crystallographic texture and accurately representing actual grains. PF rotation specifies the angles employed, when needed, to symmetrically position the orientation data. An exception is made for cases with ‘N1R’ notation, which are intentionally created with asymmetry to facilitate comparative analysis, as detailed in Section 3. Given that Kumara et al. [27] found negligible differences between low- and high-resolution EBSD maps in their analysis, this factor was not taken into account in the current study. However, it can be noted that the high-resolution EBSD sections in this work include numerous small grains, which necessitates the need for such detailed resolution. Furthermore, the studied RVE cases were modeled with three distinct thicknesses in the z-direction (BD) to assess their impact on predicting directional elastic properties, as shown in Figure 4.

Initially, panorama images across three planes—longitudinal, normal, and transversal—were captured to overview the comprehensive evolution of the microstructure and to distinguish the most representative area that struck a balance among resolution, the number of grains, and the area size, including anomaly representativeness. This step is critical in preparation for virtual testing procedures. To examine the impact in the heterogeneity of crystallographic texture, EBSD images were captured at various sample locations and directions, as seen in Figure 4. Nonetheless, further grain data implementation and tessellation focused solely on the 2D EBSD plane normal to BD with a strong <001> crystallographic texture assigned along BD. Subsequently, the plane normal to BD was selected for precise analysis and the generation of detailed 3D RVE models.

Two-dimensional EBSD grain pixel data were gathered and implemented in MTEX, which were later transformed into raster file format in Neper 4.5.0 [29]. This process incorporates resolution details, grain count, grain identity distribution matrix, and pixel orientation to generate an ASCII file format. This file then served as the basis for creating grain tessellations in the ‘tess’-file format using the ‘from_morpho’-function [30]. In this study, Voronoi tessellation, a method extensively utilized in grain tessellation, was applied to model complex grain shapes. The tessellation procedure of raster images is an intricate procedure that depends on algorithms and computational geometry to transform pixelated images into geometric shapes by mapping pixels onto a polygon mesh. Initial tessellation may not fully capture the details, particularly with complex shapes or gradients. Thus, refinement processes, like size adjustment, were undertaken to reflect the original raster image more accurately. In transitioning from 2D to 3D models, texture mapping ensures the original image visual characteristics are preserved, realistically representing the original microstructure through the detailed tessellation shown in Figure 4.

During the tessellation process, the tessellated grains are usually scaled to improve the accuracy of the grain representation matching the original microstructure. Most of the time, it might not capture the exact grain sizes and shapes due to the discretization error. The built-in scaling function in Neper facilitates the grain adjustment to represent the grain morphology more accurately, which is elaborated on in Section 3.

The dimensions of the EBSD images might influence the outcomes, where smaller resolution and fewer grains may not adequately represent the grain orientation of the entire microstructure. Therefore, the size of EBSD images were carefully chosen to capture a sufficient number of grains, as well as the anomalies of the material. Additionally, a case involving an ideal RVE, which is a synthetic depiction of a 1500-grain columnar microstructure with an ideal <001> orientation along BD, was incorporated to comprehend this influence more thoroughly.

This work, which assesses the impact on the texture breaking phenomena within the microstructure, built upon the findings from a prior study that exclusively focused on materials with strong textures [27]. Additionally, it is important to explore the extent to which virtual representations can closely approximate real microstructures and understand the role of anisotropy in the context of introducing texture-breaking and geometrical effects.

### 2.3. Computational Homogenization

Computational homogenization was used to determine the mechanical response of an RVE [31], a method particularly used to study polycrystal materials and heterogeneous structures. The RVE should contain enough information about the microstructure, such as the distribution and orientation of grains, to capture the essential features of the overall material behavior. The RVE approach is especially valuable where the material properties at a macroscale are derived from the more detailed behavior of a mesoscale. The influence of grain morphology was accounted for by solving the equilibrium equations for the entire RVE using the FE method. Virtual testing was employed to ascertain the macroscopic elastic stiffness $\bar{E}$, which characterizes the relationship between the homogenized macroscopic stress $\bar{\sigma }$ and strain $\bar{\varepsilon }$. This macroscopic elastic relation, in Voigt notation, is expressed as follows:

(2) $$\left[\begin{array}{c} {\bar{\sigma }}_{11}\\ {\bar{\sigma }}_{22}\\ {\bar{\sigma }}_{33}\\ {\bar{\sigma }}_{12}\\ {\bar{\sigma }}_{13}\\ {\bar{\sigma }}_{23} \end{array}\right]=\left[\begin{array}{c} \;{\bar{E}}_{1111}\\ \;{\bar{E}}_{2211}\\ \;{\bar{E}}_{3311}\\ \;{\bar{E}}_{1211}\\ \;{\bar{E}}_{2311}\\ {\;\bar{E}}_{1311} \end{array}\begin{array}{c} \;{\bar{E}}_{1122}\\ \;{\bar{E}}_{2222}\\ \;{\bar{E}}_{3322}\\ \;{\;\bar{E}}_{1222}\\ \;{\;\bar{E}}_{2322}\\ \;{\;\bar{E}}_{1322} \end{array}\begin{array}{c} \;{\bar{E}}_{1133}\\ \;{\bar{E}}_{2233}\\ \;{\bar{E}}_{3333}\\ \;{\;\bar{E}}_{1233}\\ \;{\bar{E}}_{2333}\\ \;{\bar{E}}_{1333} \end{array}\begin{array}{c} {\;\bar{E}}_{1112}\\ {\;\bar{E}}_{2212}\\ {\;\bar{E}}_{3312}\\ \;{\bar{E}}_{1212}\\ {\;\bar{E}}_{2312}\\ {\;\bar{E}}_{1312} \end{array}\begin{array}{c} \;{\bar{E}}_{1123}\\ \;{\bar{E}}_{2223}\\ \;{\bar{E}}_{3323}\\ \;{\bar{E}}_{1223}\\ \;{\bar{E}}_{2323}\\ \;{\bar{E}}_{1323} \end{array}\begin{array}{c} {\;\bar{E}}_{1131}\\ {\;\bar{E}}_{2213}\\ {\;\bar{E}}_{3313}\\ \;{\bar{E}}_{1213}\\ {\;\bar{E}}_{2313}\\ \;{\bar{E}}_{1313} \end{array}\;\right]\left[\begin{array}{c} {\bar{\varepsilon }}_{11}\\ {\bar{\varepsilon }}_{22}\\ {\bar{\varepsilon }}_{33}\\ {2\bar{\varepsilon }}_{12}\\ 2{\bar{\varepsilon }}_{13}\\ 2{\bar{\varepsilon }}_{23} \end{array}\right]$$

In virtual testing, we applied each component of the macroscopic strain at a time, and then the resulting homogenized stress provided a column in the stiffness matrix. Practically, this involved prescribing the displacement vector **u** on the boundary Γ of the volume element V, as follows:

(3) $$u=\bar{H}x,$$

where $\bar{H}$ represents the macroscopic displacement gradient. The components of the strain are derived from the components of $\bar{H}$**,** as follows:

(4) $${\bar{\varepsilon }}_{11}={\bar{H}}_{11},{\bar{\varepsilon }}_{22}={\bar{H}}_{22},{\bar{\varepsilon }}_{33}={\bar{H}}_{33},\;{2\bar{\varepsilon }}_{12}={\bar{H}}_{12}+{\bar{H}}_{21},2{\bar{\varepsilon }}_{23}={\bar{H}}_{23}+{\bar{H}}_{32},2{\bar{\varepsilon }}_{13}={\bar{H}}_{13}+{\bar{H}}_{31}$$

which is derived from the following relation:

(5) $$\bar{\varepsilon }=(\bar{H}+{\bar{H}}^{T})/2$$

**Figure 5:**
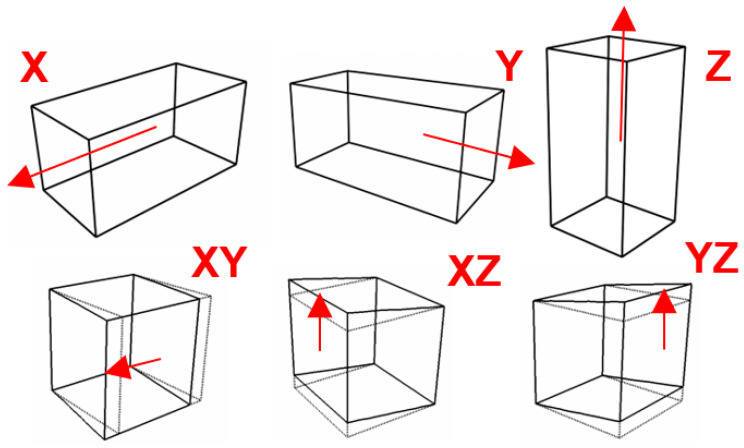
Virtual testing directions (indicated by red arrows) of an RVE.

Defining the displacement linearly is one suitable alternative when using the FE method. Figure 5 illustrates the associated virtual tests prescribing linear displacements in both the normal and shear directions.

The homogenized stress $\bar{\sigma }$ can now be computed using FE analysis as:

(6) $$\bar{\sigma }=\frac{1}{V}\sum_{e=1}^{n_{elem}}V_{e}\sigma_{e}$$

where $V_{e}$ is the element volume and $\sigma_{e}$ is the average stress in the element.

**Figure 6:**
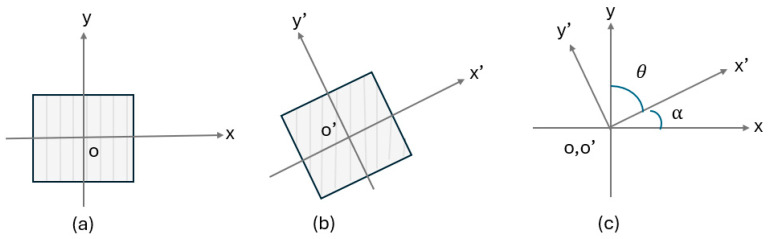
Transformation of the (**a**) xy-Cartesian coordinate system to (**b**) x’y’-Cartesian coordinate system through an angle θ and α in (**c**).

Three-dimensional CEFE simulations implementing the RVE method were performed using ABAQUS 2021 through a user material subroutine (UMAT) [32]. The RVE models were meshed with 500,000–800,000 tetrahedral second-order elements. A cubic symmetric linear elastic material model, as described in the literature [33], was implemented. In addition to grain morphology and textured orientation, the elasticity for all the grains employed the parameter values: C11 = 250.2 (GPa), C12 = 170.3 (GPa), and C44 = 100.5 (GPa) [34]. The columnar microstructure, being a standard example of a transversely isotropic material, exhibits its anisotropic behavior through five distinct elastic independent constants. In the coordinate system xoy, as illustrated in Figure 6a, the stiffness matrix is denoted by C. Consequently, by using the Voigt representation, the relation between the stress, strain, and the stiffness tensor can be expressed in the matrix format as:

(7) σ=Cε

We expressed the complete matrix as:

(8) $$\left[\begin{array}{c} \sigma_{11}\\ \sigma_{22}\\ \sigma_{33}\\ \tau_{12}\\ \tau_{13}\\ \tau_{23} \end{array}\right]=\left[\begin{array}{c} \;\;C_{11}\\ \;C_{12}\\ \;C_{13}\\ \;\;\;0\\ \;\;\;0\\ \;\;\;0 \end{array}\begin{array}{c} \;\;\;\;C_{12}\\ \;\;\;\;\;C_{22}\\ \;\;\;\;C_{23}\\ \;\;\;0\\ \;\;\;0\\ \;\;\;0 \end{array}\begin{array}{c} \;\;\;\;C_{13}\\ \;\;\;\;C_{23}\\ \;\;\;\;\;C_{33}\\ \;\;\;0\\ \;\;\;0\\ \;\;\;0 \end{array}\begin{array}{c} 0\\ 0\\ 0\\ \;\;\;C_{44}\\ 0\\ 0 \end{array}\begin{array}{c} 0\\ 0\\ 0\\ 0\\ \;\;\;\;C_{55}\\ 0 \end{array}\begin{array}{c} 0\\ 0\\ 0\\ 0\\ 0\\ \;\;\;C_{66} \end{array}\right]\left[\begin{array}{c} \varepsilon_{11}\\ \varepsilon_{22}\\ \varepsilon_{33}\\ \gamma_{12}\\ \gamma_{13}\\ \gamma_{23} \end{array}\right],$$

where $\sigma_{ij},\;\tau_{ij},\;\varepsilon_{ij},\;\mathrm{and}\;\gamma_{ij}\;$ describe the normal stresses, shear stresses, and the corresponding normal and shear strains, respectively. The transformation is depicted in Figure 6c.

In terms of the $E_{45}$ angular Young’s modulus, the element of the acquired global stiffness matrix is associated with the Young’s modulus in the direction of loading, as described by the following formula:

(9) $${E^{\prime }}_{\alpha }=C_{33}cos^{4}\left(\alpha \right)+C_{11}sin^{4}\left(\alpha \right)+\frac{C_{13}+C_{44}}{2}sin^{2}2\left(\alpha \right)$$

As authors of [27] explain, the orientation of columnar grains at various angles relative to the BD results in distinct elastic properties compared to the longitudinal and transverse directions. Furthermore, [17] reveals that variations in $C_{44}$ also influence the directional Young’s modulus. The other study by Qayyum et al. [35] similarly explores the impact of RVE thickness on the mechanical properties simulated in multi-phase materials using the CPFE method. Our study includes an analysis of combining these three effects, which are detailed in the subsequent Section 3.

## 3. Results

### 3.1. Non-Homogeneity

The AM build of different geometric shapes using PBF-EB process significantly impacts the characteristics of the material, developing distinct variations in elastic properties, as demonstrated in this study. Moreover, as EBSD data reveal, microstructural anomalies frequently occur in the produced samples. To mitigate the effects of stress concentration within the microstructure, achieving homogeneity is essential. Extensive research has been conducted to ensure optimal results. For instance, the study by Y. Wang et al. [36] suggests that real-time observation and control can be used to adjust the laser parameters and the powder layer thickness to achieve a more uniform microstructure. Meanwhile, Z. Wang et al. [37] emphasizes the influence of scanning angle on the grain growth and mechanical properties, demonstrating that varying the scanning angles can modify the thermal gradient and solidification rate during the process, which in turn influences the grain morphology and affords a more homogenized microstructure.

Given that the presence of anomalies plays a crucial role in determining the material behavior, this study extensively discusses their impact. It delves into the capabilities and limitations of accurately representative microstructures, including the non-homogeneity effects, initiating with a comparison between EBSD-generated grain data in MTEX and Neper grain tessellation regarding centroid positions, area size, and equivalent diameter distributions. The analysis progresses by examining and comparing the stiffness matrices derived from each case subjected to virtual testing. The collected stiffness matrix data were utilized to determine directional elastic properties validated against the experimental findings. The analysis encompassed distinct assessments of virtually tests accounting for the non-heterogeneity effects and later evaluated in terms of Young’s modulus, shear modulus, Poisson’s ratio, von Mises stress, and equivalent strain distributions.

An essential prerequisite to these steps involves acknowledging a key limitation in grain tessellation using Neper. Its ability to produce only convex-shaped grains results in deviations when dealing with EBSD-generated non-homogeneous microstructures, given that real grain shapes frequently display non-convex features. To repair the mismatches arising from the tessellation process and improve the alignment, scale factors were utilized, as indicated in Table 3. The increased inaccuracies observed in certain sections during tessellation can be attributed to the absence of convex grain shapes within these areas.

Convex grains can be more straightforwardly approximated by the primitives, such as triangles. In contrast, concave features or complex, non-convex shapes might not be captured accurately, leading to simplifications that do not precisely reflect the original microstructure. Another aspect to be considered is edge detection during the tessellation process. Convex grains typically have smoother, more easily identifiable edges, whereas concave or irregularly shaped grains may lead to inaccuracies in edge detection. Non-convex grains with a significant surface curvature present challenges in accurately representing the surface area. Fitting polygons to represent grain shapes in the tessellation process is more straightforward for convex grains, as the vertices of polygons can easily map to the grain outer boundaries. For non-convex or complex grain shapes, achieving a good fit requires a higher number of polygons or more sophisticated algorithms, increasing the computational complexity and inaccuracies. This study features in-depth discussions, outlining the scenarios that were explored through grain representation. It details the cases considered and provides insights into the reasoning behind their selection.

### 3.2. Grain Representation

**Figure 7:**
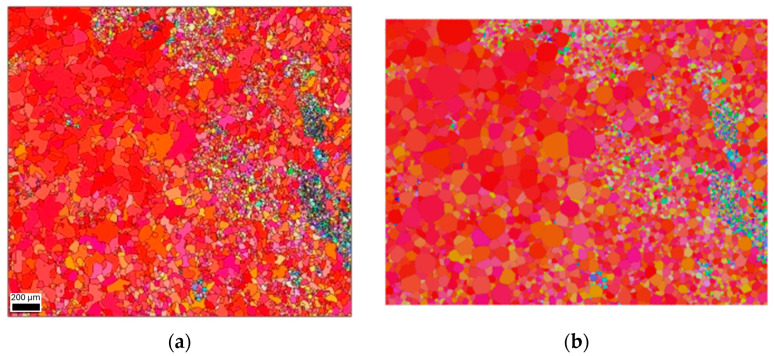
Comparison of (**a**) EBSD (Electron Backscattered Diffraction) and (**b**) the tessellation of 7000-grain microstructure in strong <001> crystallographic direction.

An example of a tessellated grain structure consisting of 7000 grains is presented in Figure 7, displaying a quantity significantly larger than typically generated in this study. Remarkably, the tessellation process proved to be highly effective for this extensive number of grains. Nevertheless, meshing such a structure demanded over 6 million elements, necessitating substantial computational power and disk space for the virtual testing of the 3D RVE. Due to these requirements, the further investigation of this section was not pursued, yet it highlighted the limitations and necessities of the specific scenarios as an example.

**Figure 8:**
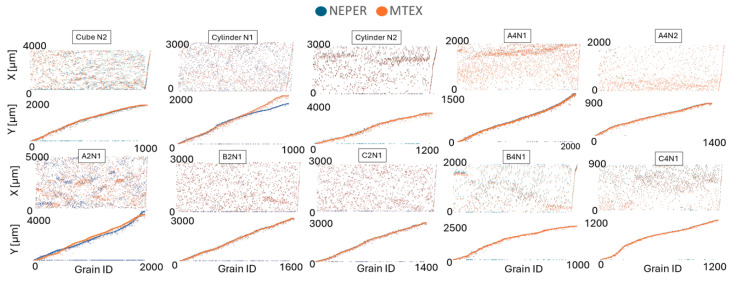
Grain centroid x and y position comparison in Neper vs. MTEX.

The selection of the EBSD sections used for virtual testing outlined in the Methodology Section receives further discussion in this section. The grain centroid positions show some significant differences for the cylinder N1 and A2N1 when compared to the measured EBSD data, as shown in Figure 8. It is important to note that, during tessellation, Neper sometimes yields a zero solution for centroid positions, indicating instances where it failed to successfully generate grains.

**Figure 9:**
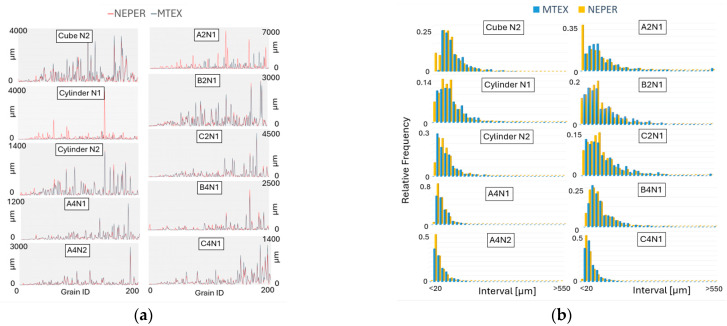
Data comparison of (**a**) a grain area of 200 grains and (**b**) grain size distributions in Neper vs. MTEX.

Neper is likely to encounter difficulties in grain tessellation in scenarios where non-convexity of grains is more pronounced. This challenge of replicating the actual grain centroid positions is evident in Figure 8, where cylinder N1 and A2N1 show the greatest deviation from the MTEX centroid position data. Similar results are observed in the comparison of the grain size area, as depicted in Figure 9a. However, this discrepancy is not reflected in the comparison of grain equivalent diameter distributions, presented in Figure 9b.

It is observed that Neper often produces a larger quantity of smaller grains compared to the grain sizes obtained from EBSD. This also indicates that the presence of non-convex grains necessitates a reduction in grain size, which is also connected to the need for implementing a scale factor during the tessellation process. Generally, the data on centroid positions and grain sizes reveal that the EBSD sections are challenging for Neper tessellation, which also correspond with the earlier presented EBSD maps, where a higher presence of non-convex shaped grains is observed. Nonetheless, the accuracy of the tessellated grain data generally shows a close correspondence with the EBSD data.

### 3.3. Stiffness Matrix

**Figure 10:**
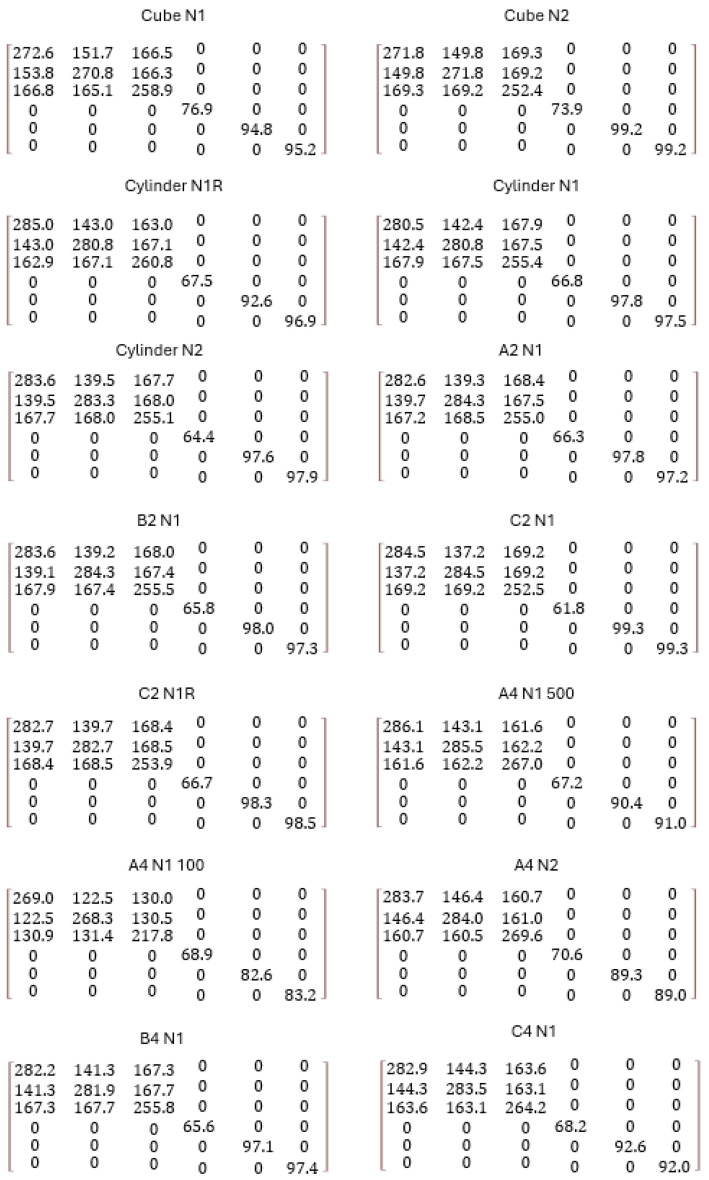
Elastic stiffness matrices of the studied RVE (Representative Volume Element) cases (unit: GPa).

Computational homogenization results in the stiffness matrix, which is crucial for solving problems related to structural deformation and stability. It is highly dependent on the material mechanical properties and the morphology as well as texture of the microstructure. In this study of anisotropic material, it can be seen how the stiffness matrix interplays with direction-dependent properties, as well as reflecting the anisotropy seen in Figure 10. The investigated cases and comparison of the stiffness matrix are a critical tool in analyzing the mechanical behavior under various loading conditions. The stress tensors owning the specific positions in the matrix present the grain orientation within the normal plane to BD. The study particularly focuses on the ND plane, which exhibits a strong texture relative to BD, also seen in Figure 10.

To generate stress tensor inequalities from the anisotropic transversal stiffness matrix, one must understand how this matrix influences stress distribution within the material. The stiffness matrix elements dictate how much strain is produced by a given stress in different directions. Since the material’s response varies with the direction, the resulting stress tensor exhibits inequalities reflecting this directional dependence as follows:

$$C_{11}=C_{22}>C_{33},\;C_{12}<C_{13}=C_{23},\;C_{44}=\frac{\left(C_{11}+C_{12}\right)}{2},C_{55}=C_{66}$$

The stiffness parallel to BD develops significantly lower than the stiffness in normal to BD. With applied load, the stress distribution within the columnar grain structure is not uniform, leading to stress tensor inequalities. These inequalities are crucial, as they help to predict failure modes and optimize the material performance under various loading conditions. Based on the performed analyses, Figure 10 shows that the set of grains and their features reinforce the specific stiffness levels linked to their orientation, size, and distribution.

### 3.4. Directional Elastic Properties

**Figure 11:**
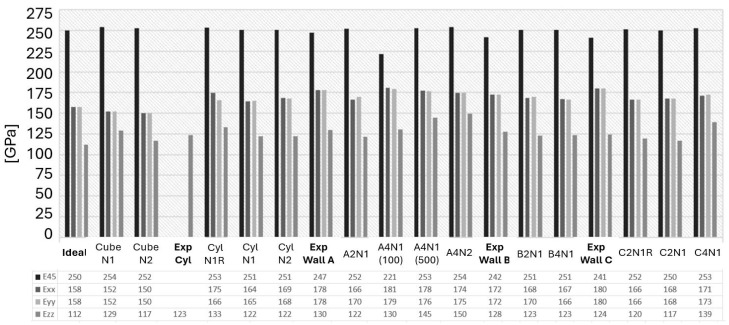
Directional Young’s modulus of the experimental and studied RVE (Representative Volume Element) cases.

**Figure 12:**
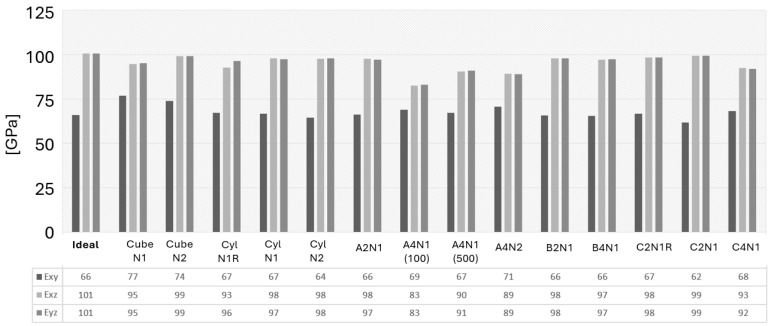
Directional shear modulus of the studied RVE (Representative Volume Element) cases.

**Figure 13:**
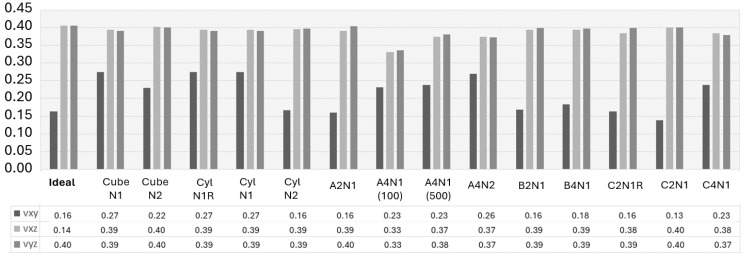
Directional Poisson’s ratio of the studied RVE (Representative Volume Element) cases.

The FE modeling predicts directional Young’s modulus, shear modulus, and Poisson’s ratio, which were validated against the experimental data, and is presented in this section, as well as shown in Figure 11, Figure 12 and Figure 13. It is observed that the selected image sizes are sufficient for predicting Young’s modulus accurately, resulting in ~5% error compared to the experimental values. It can also be noticed that there is a significant variation in the $E_{zz}$, $E_{xy}$, and $v_{xy}$ values. The stiffness sensitivity parallel to BD is rooted in the heterogeneity, which significantly impacts the present anomalies within this material. As $E_{zz}$ approaches the ideal case value of the same direction, it signifies a highly textured grain structure parallel to BD. Conversely, deviations from this value indicate an increased occurrence of texture-breaking behavior. This insight aligns with the results presented in Figure 11.

The observed A2N1, B2N1, and C2N1 cases revealed an approximately 3–5% deviation compared to the experimental data. It indicates that the selected EBSD sections lacked adequate texture-breaking effects in the microstructure. This observation underscored the challenge of capturing both a sufficient number of texture-breaking effects together with strongly textured grains within the same section. This task was deemed impractical due to the requirement for a significantly large EBSD scanning area, as exemplified in Figure 7. However, the A4N1 and A4N2 cases showed an exact alignment with the experimental data in the $E_{xx}$ and $E_{yy}$ directions. When a sufficient number of texture-braking grains are introduced, the stiffness increases in the $E_{zz}$, $E_{xz}$, and $E_{yz}$ directions, as seen in Figure 11 and Figure 12. This could also be observed in the A4N1 and A4N2 sections, where the $E_{zz}$ resulted in an overpredicted stiffness due to the lack of strongly textured grains of the investigated EBSD areas.

Figure 11 also illustrates that, in an ideal RVE case, $E_{45}$ results in a value of 250 GPa, which is roughly comparable to those seen in almost all other RVE cases of ±3 GPa variation. Yet, the average experimental value is noted to be 243 GPa in the same direction. A key discovery from the virtual testing of the A4N1 (100) case, where the RVE thickness is extensively reduced in the columnar grain direction, demonstrates a decreased $E_{45}$ along with lower shear modulus values for $E_{xz}$ and $E_{yz}$, and the Poisson’s ratio for $v_{xz}$ and $v_{yz}$, as seen in Figure 12 and Figure 13.

The decreased stiffness levels as a result from RVE thickness redaction in the A4N1 (100) case are attributed to several factors inherent to simulation and material structure behavior. The primary cause of this difference can be attributed to the scale of microstructural representation and how it interacts with the implemented boundary conditions. In a thinner volume element, the boundary conditions can have a more distinct effect on the material response due to the reduced volume and shorter path for the stress and strain distribution. This constraint can limit the ability to undergo deformation uniformly, effectively lowering its apparent shear as it reflects the resistance to shear deformation.

In the analysis of the ‘N1R’ cases, it was found that the asymmetrical rotation of the PF led to a higher correlation error when compared to the experimental observations, as illustrated in Figure 11, and produced outcomes that varied significantly from those in other cases, as depicted in Figure 12 and Figure 13. Ensuring that the grain orientation is symmetrically aligned is crucial for the accurate representation of the microstructure, which, in turn, results in predictions with a close match to the actual properties of the material. The importance of adjusting the grain orientation symmetrically is underscored as a fundamental requirement for the precise and dependable prediction of the material properties.

### 3.5. von Mises Stress Distribution

**Figure 14:**
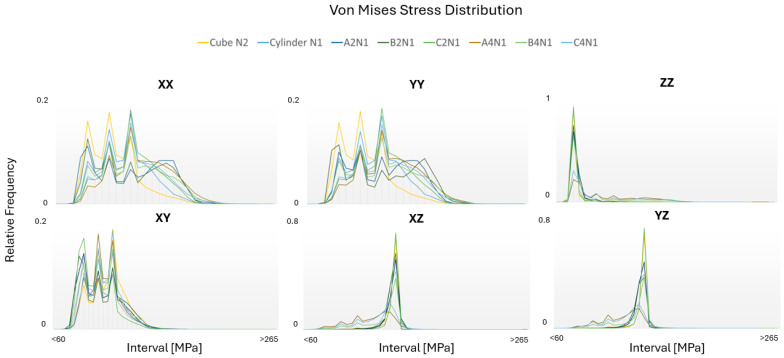
von Mises stress distributions for optimal RVE (Representative Volume Element) cases in six loading modes.

The von Mises stress is a scalar value derived from the stress tensor that represents an equivalent or effective stress that simplifies the comparison of a complex multiaxial stress state to a simple uniaxial stress. Figure 14 illustrates the von Mises stress distributions across the six tested directions of the optimal cases. It can be seen that the regions of stress concentration, differences in stress magnitudes between grains, and the influence of grain orientations on the stress localization are identified. In instances with predominantly strong textures, stress distributions exhibit more uniform tendencies resulting in a higher relative frequency, particularly in the ZZ, XZ, and YZ directions. Conversely, incorporating more texture-breaking phenomena into the microstructure leads to an increased non-uniform distribution of stress across these directions.

These insights are vital for understanding material failure mechanisms, such as crack initiation and propagation [38], and for improving material strength and toughness. Studying von Mises stress distributions offers opportunities for optimizing material properties, aiming for uniform stress distributions as the most optimized structure types. This includes selecting appropriate heat treatments [39], alloy compositions [40], or processing techniques [41] to tailor the microstructure for enhanced mechanical performance under the expected service conditions.

The distribution of the von Mises stress across the XX, YY, and XY directions reveals notable bimodal variations in the relative frequency when compared to the ZZ, XZ, and YZ directions. This uneven distribution is attributed to the randomized grain orientation within the crystal plane that is perpendicular to BD. Conversely, within the crystal plane that is parallel to BD, the columnar grain texture contributes to a significantly more uniform distribution. As the texture-breaking effects intensify, the stress distributions within those regions rise, resulting in a decreased frequency and more irregular stress distribution throughout the microstructure. Consequently, in the cube and cylinder cases, where texture-breaking effects are minimal, the frequency of stress distribution in the ZZ direction results in a nearly uniform behavior.

### 3.6. Equivalent Strain Distribution

**Figure 15:**
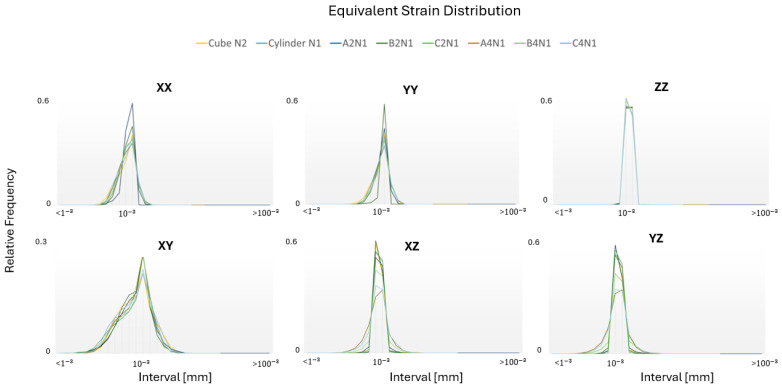
Equivalent strain distributions for optimal RVE (Representative Volume Element) cases in six loading modes.

The von Mises equivalent strains were used to assess the localized deformation within individual grains, as illustrated in Figure 15. It can be observed that the orientation of grains significantly affects the distribution of equivalent strain and the variation in the relative frequency in all directions.

The results reveal that the evaluation of the local texture is crucial since it allows for the identification of microstructural anomalies and texture variations within small regions, significantly influencing local mechanical responses.

## 4. Discussion

This paper has conducted an in-depth examination of the progress and applications of AM technologies, particularly highlighting the PBF-EB process. It has provided a detailed microstructural analysis of the Haynes^®^282^®^ alloy, emphasizing the role of the EBSD technique in analyzing the crystallographic texture, which is pivotal for generating 3D RVE models. Additionally, the capability to represent grains in 3D using 2D EBSD data simplifies the process of conducting complex analyses. The presented investigation of the grain morphology and crystallographic texture has uncovered the intricate link between microstructural features and the mechanical behaviors of anisotropic polycrystalline materials, thereby necessitating the creation of 3D RVE models, which proved to be challenging when replicating the precise grain data. The key reason for the variations in Young’s modulus values highlighted in this study is linked to irregularities in the AM material structure, a characteristic frequently observed in the PBF-EB process and associated columnar microstructures. The earlier study conducted by Kumara et al. [27] discovered that the experimentally measured Young’s modulus in the <001> direction, within a strongly textured grain structure, was around 100 (GPa), which is lower than the anticipated minimum for pure Nickel. Additionally, the investigation found that porosity, at a level of 1%, had a minimal impact on Young’s modulus and could be neglected. The study also pointed out that the precision of Young’s modulus calculations might be compromised due to the use of small tensile samples for experimental measurements conducted with Digital Image Correlation (DIC). Our work builds upon these observations by emphasizing the critical role of material anomalies, previously unconsidered, causing discrepancies in Young’s modulus measurements. It also uncovers that an increase in the prevalence of anomalies within the material correlates with enhanced stiffness and Young’s modulus in the ZZ direction. These anomalies, overlooked in past analyses, significantly influence the stiffness of the material and Young’s modulus, particularly in this direction.

This discovery prompts a revision of the assumptions made in the earlier literature, suggesting that the presence and influence of material anomalies within the strongly textured AM material were underestimated. The methodology in the reference study [27] may have overlooked the crucial role of material anomalies, primarily attributed to the discrepancy in Young’s modulus values as methodological limitations and the connected effect of porosity. Consequently, our contribution proposes that these anomalies, by increasing the material stiffness and Young’s modulus in the ZZ direction, provide a comprehensive explanation for the previously unaccounted low Young’s modulus values. This insight not only challenges prior assumptions but also highlights the complexity of accurately assessing the mechanical properties of AM materials, underscoring the need for models that incorporate the full spectrum of material characteristics, including non-homogeneity.

Employing the computational homogenization CEFE method was essential for accurately predicting elastic properties, both at the local and global scales. The depiction of von Mises stress and equivalent strain distributions provided the possibility to examine the impact of non-homogeneity on local effects. Meanwhile, the summarized results for the Young’s modulus, shear modulus, and Poisson’s ratio highlighted the global behavior of the material. Moreover, the study faced challenges with accurately representing non-convex grain shapes through tessellation, leading to the scale factor implementation to correct grain size and shape. This issue underlines the criticality of detailed EBSD area selection and the complexities involved in converting intricate microstructures into virtual models. The use of scale factors to adjust tessellated grains has highlighted challenges in directly converting non-convex grain shapes from raster images into tessellated models. This process uncovered the fact that certain EBSD sections were impractical for tessellation or meshing with Neper, owing to these specific limitations. The study emphasizes the significance of achieving a balance in EBSD area selection, including the presence of frequent anomalies in strongly textured microstructures. This selection is essential due to the impact of non-homogeneity and texture variations on the overall accuracy of the analysis. By carefully selecting EBSD areas that represent both the typical grain structure and the presence of anomalies, the analysis can capture a more realistic depiction of the overall microstructure, leading to more accurate predictions of its mechanical properties.

It also notes that discrepancies in centroid positions or grain sizes during tessellation did not significantly impact the results for elastic properties, indicating that the grain equivalent diameter distribution holds the most relevance for this type of analysis according to the findings of this study. Despite these challenges, the influence of microstructural features on the material behavior was distinctly evident. Nevertheless, it underscores that adjusting for the asymmetrical grain orientation within the selected microstructure is essential to achieve accurate predictions of material properties.

The significance of the RVE thickness in influencing the directional property prediction accuracy is emphasized by findings that demonstrate a substantial decrease in Young’s modulus values in the $E_{45}$ direction. This thickness reduction is associated with a compromised shear capability of the material when faced with stress from this specific angle. The employment of thinner RVEs reduces the ability to adequately distribute and mitigate shear forces. Such observations underscore the criticality of considering RVE thickness for accurate predictions of material mechanical behavior.

## 5. Conclusions

The findings from this study highlighted the capability of the developed virtual models to predict directional elastic properties with a high degree of accuracy. This achievement underscores the potential of the proposed methodology for enhancing predictive modeling capabilities in the AM field, especially for materials characterized by complex, non-homogeneous microstructures. Nevertheless, accurately representing non-convex grain shapes exhibited challenges, necessitating the accurate selection of EBSD areas due to variations in grain size and shape. Despite these challenges, the study illustrated the impact of microstructural features on material behavior, emphasizing the critical role of detailed EBSD area selection and the intricate process of transforming complex microstructures into virtual models.

Future activities will aim to refine the accuracy of virtual models that mimic microstructures, tackle the representation of non-convex grains, and determine an optimal balance in EBSD area selection for more precise and predictive modelling outcomes. This becomes especially critical in the context of other AM processes, like PBF-LB, as well as in regions where diverse grain structures intersect, affecting the material behavior locally within the interfaces. The continuous improvement in microstructural analysis techniques is expected to enhance our comprehension of material properties and stress distribution, both at the macroscale and locally within the grains, where achieving exact fidelity to the actual microstructure becomes increasingly crucial.

## Figures and Tables

**Table 1:** Chemical composition of the Haynes^®^282^®^ powder standardized as [26] 700.

Ni (wt%)	Cr (wt%)	Co (wt%)	Mo (wt%)	Ti (wt%)	Al (wt%)	C (wt%)	O (ppm)	N (ppm)
Balance	18.9	9.5	8.3	2.0	1.44	0.07	<250	<250

**Table 2:** Implemented key process parameters.

EBMC Version	Beam Current	Beam Focus Offset	Beam Velocity	Line Offset	Scan Length
6.1.15	15 mA	20 mA	2500 mm s^−1^	0.125 mm	40 mm

**Table 3:** Scale factors applied to grain attributes during the tessellation process.

Section	Centroid X	Centroid Y	Grain Equivalent Diameter	Grain Size
Cube N2	-	-	-	0.1
Cylinder N1	2.5	0.33	-	0.04
Cylinder N2	-	-	-	0.077
A2N1	1.3	0.8	1.54	0.0625
B2N1	-	-	0.1	0.0625
C2N1	-	-	-	0.0625
A4N1	-	-	-	0.1
A4N2	-	-	-	0.1
B4N1	-	-	-	0.022
C4N1	-	-	-	0.1

## Data Availability

Data are contained within the article. The raw data supporting the conclusions of this article will be made available by the authors on request.

## References

[1] Attallah M.M., Jennings R., Wang X., Carter L.N. (2016). Additive manufacturing of Ni-based superalloys: The outstanding issues. MRS Bull..

[2] Zhang L.-C., Liu Y., Li S., Hao Y. (2017). Additive Manufacturing of Titanium Alloys by Electron Beam Melting: A Review. Adv. Eng. Mater..

[3] Parimi L.L. (2014). Additive Manufacturing of Nickel Based Superalloys for Aerospace Applications. Ph.D. Thesis.

[4] Tan X., Kok Y., Tan Y.J., Descoins M., Mangelinck D., Tor S.B., Leong K.F., Chua C.K. (2015). Graded microstructure and mechanical properties of additive manufactured Ti–6Al–4V via electron beam melting. Acta Mater..

[5] Alami A.H., Olabi A.G., Alashkar A., Alasad S., Aljaghoub H., Rezk H., Abdelkareem M.A. (2023). Additive manufacturing in the aerospace and automotive industries: Recent trends and role in achieving sustainable development goals. Ain Shams Eng. J..

[6] Unocic K., Kirka M., Cakmak E., Greeley D., Okello A., Dryepondt S. (2019). Evaluation of additive electron beam melting of haynes 282 alloy. Mater. Sci. Eng. A.

[7] Jablonski P.D., Hawk J.A., Cowen C.J., Maziasz P.J. (2012). Processing of Advanced Cast Alloys for A-USC Steam Turbine Applications. JOM.

[8] Bernier J.V., Suter R.M., Rollett A.D., Almer J.D. (2020). High-Energy X-Ray Diffraction Microscopy in Materials Science. Annu. Rev. Mater. Res..

[9] López J.G., Kestens L.A.I. (2021). A multivariate grain size and orientation distribution function: Derivation from electron backscatter diffraction data and applications. J. Appl. Crystallogr..

[10] Quey R., Renversade L. (2018). Optimal polyhedral description of 3D polycrystals: Method and application to statistical and synchrotron X-ray diffraction data. Comput. Methods Appl. Mech. Eng..

[11] Pauza J., Tayon W.A., Rollett A.D. (2021). Computer simulation of microstructure development in powder-bed additive manufacturing with crystallographic texture. Model. Simul. Mater. Sci. Eng..

[12] Vuppala A., Krämer A., Lohmar J. (2021). On Sampling Discrete Orientations from XRD for Texture Representation in Aggregates with Varying Grain Size. Crystals.

[13] Lizarazu J., Harirchian E., Shaik U.A., Shareef M., Antoni-Zdziobek A., Lahmer T. (2023). Application of machine learning-based algorithms to predict the stress-strain curves of additively manufactured mild steel out of its microstructural characteristics. Results Eng..

[14] Calcagnotto M., Ponge D., Demir E., Raabe D. (2010). Orientation gradients and geometrically necessary dislocations in ultrafine grained dual-phase steels studied by 2D and 3D EBSD. Mater. Sci. Eng. A.

[15] Hermann W., Sockel H., Han J., Bertram A. (1996). Elastic Properties and Determination of Elastic Constants of Nickel-Base Superalloys by a Free-Free Beam Technique. Superalloys.

[16] Zaikovska L., Ekh M., Kumara C. Virtual Testing of Synthetic Polycrystal Microstructures Predicting Elastic Properties of Additive Manufactured Alloy 718. Proceedings of the 9th World Congress on Mechanical, Chemical, and Material Engineering (MCM’23).

[17] Wang H., Zhou H., Gui L., Ji H., Zhang X. (2014). Analysis of effect of fiber orientation on Young’s modulus for unidirectional fiber reinforced composites. Compos. Part B Eng..

[18] Biswas A., Prasad M.R.G., Vajragupta N., Kostka A., Niendorf T., Hartmaier A. (2020). Effect of Grain Statistics on Micromechanical Modeling: The Example of Additively Manufactured Materials Examined by Electron Backscatter Diffraction. Adv. Eng. Mater..

[19] Mandal S., Pradeep K., Zaefferer S., Raabe D. (2014). A novel approach to measure grain boundary segregation in bulk polycrystalline materials in dependence of the boundaries’ five rotational degrees of freedom. Scr. Mater..

[20] Cazic I., Zollinger J., Engstler M., Ghanbaja J., Schenk T., El Kandaoui M., Appolaire B. (2023). Nucleation burst in additively manufactured Inconel 718: 3D characterization of ISRO-induced equiaxed microstructure. Addit. Manuf..

[21] Wormser C. (2009). Generalized Voronoi Diagrams and Applications. Ph.D. Thesis.

[22] Zheng X., Sun T., Zhou J., Zhang R., Ming P. (2022). Modeling of Polycrystalline Material Microstructure with 3D Grain Boundary Based on Laguerre–Voronoi Tessellation. Materials.

[23] Balanovskiy A.E., Astafyeva N.A., Kondratyev V.V., Karlina A.I. (2021). Study of mechanical properties of C-Mn-Si composition metal after wire-arc additive manufacturing (WAAM). CIS Iron Steel Rev..

[24] Balanovskiy A.E., Astafyeva N.A., Kondratyev V.V., Karlina Y.I. (2022). Study of impact strength of C-Mn-Si composition metal after wire-arc additive manufacturing (WAAM). CIS Iron Steel Rev..

[25] Karlina A.I., Karlina Y.I., Kondratiev V.V., Kononenko R.V., Breki A.D. (2023). Study of Wear of an Alloyed Layer with Chromium Carbide Particles after Plasma Melting. Crystals.

[26] EOS (2022). EOS NickelAlloy HAYNES ® 282 ® Material Data Sheet. https://www.eos.info.

[27] Kumara C., Deng D., Moverare J., Nylén P. (2018). Modelling of anisotropic elastic properties in alloy 718 built by electron beam melting. Mater. Sci. Technol..

[28] “MTEX”. https://mtex-toolbox.github.io/.

[29] “Neper”. https://neper.info/doc/index.html.

[30] Quey R. (2020). Neper Reference Manual.

[31] Segurado J., Lebensohn R.A., Llorca J. (2018). Computational Homogenization of Polycrystals. Adv. Appl. Mech..

[32] Writing User Subroutines with ABAQUS, “Lecture 6 Writing a UMAT or VUMAT,” pp. 1–117. https://www.google.se/url?sa=t&rct=j&q=&esrc=s&source=web&cd=&ved=2ahUKEwioi7LI4qaFAxUrFhAIHZrbCkcQFnoECA8QAQ&url=https%3A%2F%2Fimechanica.org%2Ffiles%2FWriting%2520a%2520UMAT.pdf&usg=AOvVaw3TyaLZWkIVT7x3ilnlr29m&opi=89978449.

[33] Benedetti I., Barbe F. (2013). Modelling Polycrystalline Materials: An Overview of Three-Dimensional Grain-Scale Mechanical Models. J. Multiscale Model..

[34] Goulas A., Southcott-Engstrøm D., Friel R.J., Harris R.A. Solid Freeform Fabrication 2016. Proceedings of the 26th Annual International Solid Freeform Fabrication Symposium—An Additive Manufacturing Conference Reviewed Paper.

[35] Qayyum F., Chaudhry A.A., Guk S., Schmidtchen M., Kawalla R., Prahl U. (2020). Effect of 3D Representative Volume Element (RVE) Thickness on Stress and Strain Partitioning in Crystal Plasticity Simulations of Multi-Phase Materials. Crystals.

[36] Wang Y., Guo W., Xie Y., Li H., Zeng C., Xu M., Zhang H. (2024). In-situ monitoring plume, spattering behavior and revealing their relationship with melt flow in laser powder bed fusion of nickel-based superalloy. J. Mater. Sci. Technol..

[37] Wang Z., Yang Z., Liu F., Zhang W. (2023). Influence of the scanning angle on the grain growth and mechanical properties of Ni10Cr6W1Fe9Ti1 HEA fabricated using the LPBF–AM method. Mater. Sci. Eng. A.

[38] Simonovski I., Cizelj L. (2015). Cohesive zone modeling of intergranular cracking in polycrystalline aggregates. Nucl. Eng. Des..

[39] Jiang R., Mostafaei A., Pauza J., Kantzos C., Rollett A.D. (2019). Varied heat treatments and properties of laser powder bed printed Inconel 718. Mater. Sci. Eng. A.

[40] Agius D., O’toole P., Wallbrink C., Sterjovski Z., Wang C.-H., Schaffer G.B. (2021). Integrating phase field and crystal plasticity finite element models for simulations of titanium alloy Ti-5553. J. Phys. Mater..

[41] Karimi P., Sadeghi E., Ålgårdh J., Olsson J., Colliander M.H., Harlin P., Toyserkani E., Andersson J. (2021). Tailored grain morphology via a unique melting strategy in electron beam-powder bed fusion. Mater. Sci. Eng. A.

